# Psychiatric comorbidities of median arcuate ligament syndrome: Indications for intervention across the lifespan

**DOI:** 10.46439/psychiatry.1.006

**Published:** 2021

**Authors:** Estée CH Feldman, Collen Stiles-Shields, Grace Zee Mak, Christopher L Skelly, Tina Drossos

**Affiliations:** Department of Psychiatry and Behavioral Neuroscience, The University of Chicago Medicine and Biological Sciences, Chicago, IL, USA

Median Arcuate Ligament Syndrome (MALS) is the terminology that describes the vascular compression of the celiac artery [1], which at times is associated with numerous gastrointestinal symptoms. Most notably, patients with MALS present with epigastric pain, often worsening post-prandially, and weight loss [2]. Despite the often-striking symptom presentation of patients, significant hesitation in treating MALS is present, partially due to the lack of understanding of the pathophysiological mechanism of pain in this condition [3,4]. For example, while up to 50% of the general population presents with radiographic features of celiac artery compression [5], a much smaller percent reports the aforementioned symptoms consistent with clinical features of MALS. As such, some physicians question the validity of the diagnosis (e.g., Szilagyi et al., [5]), and suggest that it necessitates “high clinical suspicion” [6]. Others suggest that rather than a distinct diagnosis, individuals presenting with MALS may simply be presenting with idiopathic gastroparesis [7]. The lack of physician recognition and understanding of MALS may contribute to invalidating clinical encounters for patients, as well as increased time to diagnosis and treatment [8].

The diagnosis of MALS is further complicated by the high incidence of psychopathology noted in this disease population. As previously reported by Skelly and colleagues [9], nearly 28% of adults presenting for corrective surgery demonstrated symptoms consistent with a DSM-IV-TR psychiatric diagnosis (see Mak et al. [1] and Skelly et al. [9] for detailed explanations of the surgical procedure). More specifically, 16% presented with symptoms consistent with a diagnosis of an anxiety disorder, while 7.8% presented with a mood disorder, 11.8% with an adjustment disorder, and 3.9% with attention-deficit/hyperactivity disorder. Notably, such a pattern of increased psychiatric comorbidity presents in pediatric populations with MALS as well, suggesting that patients with MALS present with a lifelong increased incidence of psychiatric diagnoses. Among pediatric patients similarly presenting for surgical correction of MALS, 43% presented with symptoms consistent with a DSM-IV-TR psychiatric diagnosis [1]. As in adult populations, anxiety disorders were most commonly reported (23% of sample), with fewer participants meeting criteria for a mood disorder (13.3%), an adjustment disorder (13.3%), or attention-deficit/hyperactivity disorder (6.7%). When levels of depression and anxiety were compared to community samples of children with inflammatory bowel disease and functional gastrointestinal diseases, children and adolescents with MALS indicated average scores comparable to the two aforementioned disease groups. This finding indicated that incidence of psychiatric symptoms is not only higher than in the general population, but is comparable to other disease groups who frequently experience abdominal pain. Given the increased incidence of psychiatric symptoms and diagnoses among both adult [9] and pediatric [1] populations with MALS, consideration of the cause of increased incidence of psychopathology is necessary.

A diathesis-stress model of psychopathology in the context of chronic pain suggests that pain and GI symptoms associated with MALS may trigger, or be triggered by, mental health concerns [10]. Among patients with pain in the context of MALS, stressful and frequently invalidating experiences with healthcare providers may interact with negative schemas and attributional style diatheses to foster the development of psychopathology. Such interactions with providers, frequently marked by: 1) messages conveying physician inability to detect cause of epigastric pain; 2) suggestions of somatizations of pain; or 3) being unsure of what to do to alleviate said pain, function as rejecting and invalidating responses. This rejection and invalidation may activate cognitive distortion processes, leading to distrust of medical providers more broadly, and potential feelings of hopelessness, lack of control and depressogenic attributions for pain [11]. Furthermore, as a result of medical distrust, patients may instead turn to use of external resources (e.g., internet, social media), which while with positive aspects (e.g., access to support groups and education), may also scaffold the development of negative cognitive consequences *via* introduction of unfounded theories and maladaptive group-catastrophizing of symptoms. In turn, the reciprocal relationship between such cognitions and the perception of pain contributes to the maintenance of psychopathology and increased severity of chronic pain. Ultimately, the perpetuation of this cycle yields the development of full psychiatric syndromes [10]. While initially described in the context of depressive disorders, similar diathesis-stress models may account for elevated incidents of other forms of psychopathology, such as anxiety symptoms and disorders, as are frequently endorsed by this population [1,9,12].

Such a diathesis-stress model for the relationship between chronic pain and psychiatric symptoms is supported in other populations, wherein elevated rates of psychopathology are described in the context of numerous adult and pediatric chronic pain populations. For example, within a community sample of adults with various forms of pain, including abdominal pain, a relationship between self-reported chronic pain and mild and major depressive symptoms was noted such that those with chronic pain were more likely to endorse these depressive symptoms than healthy controls (25% *vs.* 5%; Kawai et al., [13]). Such relationships between psychopathology and chronic pain are not limited to depression. In a study of adults with IBD, pain presents as one of the strongest correlates of anxiety, wherein those with chronic pain were 2.43 times more likely to experience anxiety than those without pain [14]. Moreover, other lines of inquiry have noted that in adults with chronic pain, as anxiety symptoms increase, the likelihood of experiencing disabling pain similarly increases [15]. This is consistent with Banks and Kerns’ theory that psychiatric symptoms and pain interact with one another to produce more profound pathology and greater resultant deficits in psychosocial functioning (i.e., greater disability due to pain) [10]. Similar relationships are reported in pediatric populations. Indeed, children and adolescents who present with chronic pain report higher levels of anxiety and greater depression in the context of higher levels of pain [16]. Furthermore, adults who experienced chronic pain in adolescence report significantly higher lifetime rates of anxiety disorders (21.1% *vs.*12.4%) and depressive disorders (24.5% *vs.*14.1%) as compared to individuals without a history of adolescent chronic pain [17]. As such, there appear to be lifelong effects from the experience of pain, necessitating early, effective intervention to protect future psychosocial functioning.

Given the association of poorer clinical outcomes for adults [9,18] and pediatric [19] patients with MALS with psychiatric symptoms and disorders, evaluation and implementation of treatment for such disorders prior to surgery is crucial. As pain may contribute to the development and maintenance of psychiatric symptoms in this population, interventions aimed at addressing psychopathology must consider and account for the role of pain in the etiopathology. For example, psychological interventions would benefit from being tailored to directly address pain and related coping strategies. Evidence in chronic pain populations more generally supports this, wherein cognitive behavioral therapy (CBT) is regarded as the “gold standard” of psychological interventions for chronic pain, with efficacy shown for reduction of pain, distress, pain interference with activities, and disability [20]. In addition to alleviating disability immediately post treatment, CBT has demonstrated persistent therapeutic effects, with reduced depression at 6-months post treatment [21]. As such, cognitive behavioral interventions tailored to address pain in the context of MALS, with consideration of unique stressors associated with such a diagnosis (e.g., lack of widespread and physician understanding of illness, dismissal of symptoms) may be best suited to address MALS-related epigastric pain, and often related psychiatric symptoms, and should be tested in a prospective trial.
